# Qualitative Classification of Late Systemic Symptoms in Head and Neck Cancer Survivors

**DOI:** 10.3390/cancers16112106

**Published:** 2024-05-31

**Authors:** Poppy Schoenberg, Elizabeth Wulff-Burchfield, David Schlundt, Kemberlee Bonnet, Mary Dietrich, Barbara Murphy

**Affiliations:** 1 Osher Center for Integrative Health, Vanderbilt University Medical Center, Nashville, TN 37203, USA; 2 Department of Medicine, University of Kansas Medical Center, Kansas City, KS 66160, USA; 3 Department of Psychology, Vanderbilt University, Nashville, TN 37212, USA; 4 Department of Biostatistics, School of Nursing, Vanderbilt University, Nashville, TN 37240, USA; 5 Department of Medicine, Vanderbilt University Medical Center, Nashville, TN 37203, USA

**Keywords:** systemic symptoms, head and neck cancer, cancer survivorship, emotional disturbance, fatigue, pain, social support, spirituality

## Abstract

**Simple Summary:**

Despite advances in head and neck cancer control, survivors encounter significant difficulties accessing survivorship care in the USA. We conducted a qualitative study aimed to better understand their experiences and identify unmet needs. Fifteen long-term head and neck cancer survivors were interviewed, where themes around chronic emotional distress, fatigue, and disruptions in daily life emerged across the focus group. Secondary issues included sleep problems, cognitive difficulties, and other health conditions. Surprisingly, physical symptoms like pain and changes in appetite were less discussed. These findings underscore the need for tailored holistic multi-dimensional cancer survivorship programs that address physical, emotional, and spiritual well-being. The study highlights the importance of increased awareness and comprehensive long-term support to prioritize and enhance quality of life for the head and neck cancer survivorship community.

**Abstract:**

Improved rates of cancer control have increased the head and neck cancer survivor population. Cancer survivorship clinics are not widely available in the USA, and longitudinal supportive care for patients undergoing multimodal therapy has not advanced at a pace commensurate with improvements in cancer control. Consequently, a large head and neck cancer survivor population whose quality of life may be chronically and/or permanently diminished presently exists. This lack of awareness perpetuates under-recognition and under-investigation, leaving survivors’ (mostly detrimental) experiences largely uncharted. We conducted a qualitative exploration of survivors’ experiences, aiming to unpack the profound impact of late systemic symptoms on daily life, encompassing work, relationships, and self-identity in the head and neck cancer survivor community. The study included 15 remitted head and neck survivors, ≥12 months from their final treatment, who participated in semi-structured interviews conducted by a medical oncologist. Data analysis comprised qualitative thematic analysis, specifically inductive hierarchical linear modeling, enriched by a deductive approach of anecdotal clinical reporting. Results highlighted that 43.36% of all quotation material discussed in the interviews pertained to chronic emotion disturbance with significant implications for other domains of life. A central symptom cluster comprised impairments in mood/emotions, daily activity, and significant fatigue. Dysfunction in sleep, other medical conditions, and cognitive deficits comprised a secondary cluster. Physical dysfunctionality, encompassing pain, appetite, and eating, and alterations in experienced body temperature, constituted a tertiary cluster, and perhaps were surprisingly the least discussed symptom burden among head and neck cancer survivors. Symptoms causing heightened long-term survivor burden may be considered epiphenomenal to central physical dysfunctionality, albeit being presently the least represented in cancer survivor care programs. Moving forward, the development of targeted and multi-dimensional treatment programs that encompass physical, psychosocial, and spiritual domains are needed to increase clinical specificity and effective holistic long-term solutions that will foster a more compassionate and informed future of care for the cancer survivorship community.

## 1. Introduction

Head and neck cancer ranks as the eighth most prevalent cancer worldwide, and is predicted to be responsible for more than 58,000 newly diagnosed cases in the United States in 2024 [1]. More than two-thirds of new head and neck cancer cases present at a locally advanced stage, for which multimodality treatment including surgery, chemotherapy, and/or radiotherapy has resulted in improved disease control and survival, constituting the standard of care [2]. Owing to improved rates of cancer control, the head and neck cancer survivor population continues to grow at an unprecedented rate [3]. While the term cancer “survivor” is defined variably in the literature and by patient advocacy organizations [4], both sub-populations, i.e., patients living with, and after head and neck cancer, are demonstrating substantial growth [3]. Supportive care for acute treatment toxicities has improved meaningfully over the past two decades [5,6], although longitudinal supportive care for patients undergoing multimodal therapy is presently not advancing at a pace commensurate with improvements in acute symptom management or cancer control. This has resulted in a growing cancer survivor population whose quality of life may be chronically, and in many cases permanently, diminished [7,8,9,10,11,12].

Chronic toxicities and symptoms from head and neck cancer and/or its therapy may include locoregional side effects such as adverse dental effects or tooth loss [13,14], lymphedema [15], swallowing dysfunction [16], pain [17,18], and other localized suffering. However, more generalized symptoms include, but are not limited to, fatigue, thermal discomfort, appetite problems, sleep disturbance, and mood disorders, which are considered systemic in nature [19]. Both local and systemic symptoms can be noted prior to, during, or even months after completing cancer treatment, and both local and generalized/systemic adverse effects may persist months or even years longer than previously recognized [20,21]. Whilst novel interventions are being developed and implemented for the mitigation of, and prophylaxis for, local toxicities [22,23,24,25,26], the lack of published data characterizing chronic systemic symptoms has hindered meaningful research into management and prevention strategies [27]. It has been noted that late systemic symptoms tend to occur in clusters [28], suggesting a common underlying pathobiology. However, a comprehensive investigation into the mechanisms and pathways contributing to systemic symptoms has yet to be undertaken. Preliminary evidence suggests that inflammation [29], specifically, pro-inflammatory cytokines instigated by the native immune response or treatment effects from head and neck cancer treatment, may result in a state of chronic, centralized inflammation and chronic, systemic symptom burden—a hypothesis that has yet to be fully developed [30,31,32]. Recently, we presented the first data supporting such a proof-of-concept with scope for all non-central nervous system cancers, including the head and neck cancer sub-population. Our new data found increased microglial activation in long-term head and neck cancer survivors, versus matched healthy controls, that was specifically linked to chronic systemic symptomatology [33]. These data were particularly interesting given that other markers of inflammation and neurodegenerative decline, such as peripheral blood inflammatory markers or neurovascular measures of white tract integrity, did not correlate with chronic systemic symptoms [33], supporting a peripheral-to-centralized neuroinflammatory basis of such symptomatology.

The focus of this report pertains to the general lack of clinician and investigator awareness of systemic symptomatology, which (a) serves to perpetuate the under-recognition and under-investigation of meaningful innovation in this domain of clinical study and care, and (b) allows head and neck cancer survivors’ experiences and struggles to be largely medically and experientially unchartered. To the best of our knowledge, there are no published comprehensive qualitative data regarding the extent and impact of these experiences among head and neck cancer survivors. To address this gap in the knowledge, we conducted a qualitative study, aiming to unravel the head and neck cancer survivor experience and the profound impact of late systemic symptoms on daily life, encompassing work, relationships, and self-identity. A driving goal of this study was to amplify survivors’ voices, empower the survivorship community, and foster a more compassionate and informed future for head and neck cancer survivorship support. By including a diverse sample of survivors from various socio-economic backgrounds and catchment areas, we sought to better understand the unique challenges and needs of these individuals, ultimately paving the way for more equitable and effective survivorship care programs.

## 2. Materials and Methods

### 2.1. Sample and Recruitment

Fifteen patients (eleven male, four female) were recruited from the Henry Joyce Cancer Clinic at the Vanderbilt-Ingram Cancer Center between November 2014 and November 2016, a subset from a wider cohort of 105 enrolled in a clinical trial, for the reported qualitative analysis. The qualitative study was approved by the Institutional Review Board and Scientific Review Committee at Vanderbilt University, and all patients provided signed informed consent prior to interviews. Potential patients were identified through review of medical records and were eligible to participate in the qualitative interview if they met the following criteria: (1) ≥21 years of age, (2) at least one moderate to severe systemic symptom, (3) prior histologic diagnosis of head and neck cancer, (4) ≥12 months after completing all head and neck cancer treatment, (5) no current evidence of cancer, (6) able to speak English, and (7) able to provide informed consent.

### 2.2. Data Collection

Interviews were semi-structured and ranged from 10 to 70 min. The interview guide was developed from the medical consensus and specific questions from head and neck cancer healthcare providers. Interviews elicited information about patients’ chronic systemic symptoms during and following their head and neck cancer treatment. Questions encompassed multiple domains including quality of life, interpersonal relationships, professional functioning, and identity. Follow-up questions were asked for clarity and to promote detailed discussion of survivors’ experiences. All interviews were conducted by the same trained interviewer (EWB), recorded, and transcribed (www.Rev.com (accessed on 17 May 2024)) prior to de-identification. Transcripts were reviewed and checked for quality assessment by EWB and another trained staff member (BAM) who was not involved in either interviewing patients or the transcription process. Data saturation was determined by the research team and recruitment of new patients ceased once data saturation was reached. Data saturation comprised a judgement as to whether or not new information was emerging from the most recent interviews.

### 2.3. Data Analysis

Transcribed interviews were analyzed by the Vanderbilt University Qualitative Research Core using thematic analysis. Data coding and analysis were led by a PhD-level psychologist (DGS) following COREQ guidelines [34], and implemented by a trained qualitative researcher (KRB). A hierarchical coding system was developed and refined using the interview guide and a preliminary review of the transcripts. Definitions and rules were written for the use of each category. Five major categories were identified, which were further branched into fourteen subcategories, some with additional hierarchical divisions. The final coding system included the following 16 overall categories: (1) Fatigue; (2) Sleep; (3) Cognition; (4) Body Temperature; (5) Emotions; (6) Appetite and Food; (7) patient’s Medical Condition; (8) Aches and Pain; (9) Daily Activities; (10) Relationships; (11) Symptom Improvement; (12) Declining Trajectory; (13) Delayed Symptom Onset; (14) Disability; (15) Coping Strategies; and (16) Spirituality. See Supplementary Materials: (https://healthbehavior.psy.vanderbilt.edu//Wulff/Wulff_CodingSystem.pdf (accessed on 17 May 2024)).

Each statement was treated as a separate quotation, with each quotation assigned up to six codes. Inter-rater reliability was established by two investigators separately coding transcripts, reconciling their codes, and establishing inter-rater consensus. Codes were then analyzed using an iterative inductive–deductive approach. The deductive approach was guided by significant clinical experience in caring with head and neck cancer patients, in addition to the research literature on cancer symptoms and quality of life. Inductively, we sorted the codes in statistical software to synthesize the data into higher-order themes using hierarchical linear modeling.

## 3. Results

Emotions were the most frequently discussed symptoms by patients as a whole (43.36%), followed by Daily Activities (32.16%) and Fatigue (31.64%). Table 1 outlines the mean percentage of quotations for each category, which were also calculated when weighting the individual data quota to the full sample. Exemplary quotations extracted from the interview data are further reported below, and in Table 2. Only categories discussed in ≥10% of interview data across the patient pool are discussed.

### 3.1. Mood and Emotion Dysregulation

The most common long-term burden, experienced by all patients, pertained to emotions as opposed to direct physical symptoms, constituting 43.26% of discussions during the interviews. Naturally, stress became a significant burden; dealing with symptoms and the threat of cancer returning all served to amplify the stressors of everyday living: “*It’s like I can’t handle what I used to could handle. I used to could handle a whole lot of stress and I used to could handle pressure…I cannot now [sic]*”.

Feelings of anxiety were commonly reported, with some patients reporting anxiety around specific triggers, and others a generalized anxiety, that further compacted other dimensions of symptomatology. Sudden onset of anxiety was also reported that was not necessarily related to fear of cancer: “*I’ve had some problems with anxiety…One of the nice things about being so ill is I’m not afraid of dying…I don’t want to do is to be disabled and live a long time.*”; but longer term consequences from treatments; “*…my anxiety towards eating, swallowing and going grocery shopping that’s cause the worst anxiety throughout whole day now [sic]…I’ll have an episode…It’s all tied into my eating and choking*”.

Depression was common, with generally an insidious onset: “*I think that the depression started gradually over the following year…I didn’t just wake up one day tired and depressed. It was a slow, gradual thing that occurred about six months after treatment*”.

Some patients reported severe and intense depression: “*I think about killing myself*”, “*Yes, depression is pervasive*”, “*[depression] it impacts me on a daily basis*”.

Depressive symptoms would limit help seeking and social support behaviors causing further detriment: “*If I am depressed, I really don’t want to associate with anybody [inaudible 00:37:37]*”.

The misfortune of both anxiety and depression also persisted longer term: “*[depression and anxiety]…been gradually getting worse for the last 8 or 10 years, since I had first gotten cancer*”.

Emotional dysfunction had a detrimental impact on self-esteem and identity, such as: “*It’s played a large role in my self-worth, because as a guy not working, it’s wiped out a lot of my self-worth*”, “*Sometimes I don’t feel worthy.*”, “*I went on disability which did the transition for me just mentally to think that I’m disabled, and that has been part of the whole depression thing too*”, and “*It makes me feel less of a person that I used to be*”.

### 3.2. Daily Activities

Significant burden around daily activities was the second most discussed. Cumulatively, symptoms severely impacted professional, social, interpersonal, and recreational activities. Being able to continue in employment, or conduct their work properly, was a major issue for many patients: “*I couldn’t work 40 h for anyone I know that, but I’m learning how to manage it better*”.

Hindrance in the simplest of daily physical activities were also reported due to lack of vigor: “*I’m not as strong as I was … like this morning, opening up a box of cereal in here and trying to pull the cereal itself, the plastic, apart. I can get it, but it’s pretty tough.*”, and “*I’ve had several [inaudible 00:09:35] a bath because I’m not strong enough to pull myself out of the bathtub.*”, and “*If I walk across the yard and back I have to set down. I put it that way.*”, further to mobility issues; “*It really affects my ability to drive, that’s why my wife do a lot of driving. I don’t have the full range of motion that I want to turn my neck from side to side*”.

### 3.3. Fatigue and Sleep

Fatigue was the third most common burden discussed by 14 patients, and sleep challenges were the fourth more commonly discussed issue experienced by all patients. Sleeping problems and fatigue were believed to be “*brought on by treatments*”, “*…the chemo radiation*”, but didn’t dissipate once treatment ceased: “*I kept sleeping more and more after cancer treatment, so a year after cancer treatment is when I was sleeping fifteen hours a day, and it wasn’t insomnia*”.

Treatment sometimes damaged other organs: “*My thyroid was damaged, so I’m a thyroxine [sic]*”, causing additional long-term complications for metabolism and sleep.

A large portion of patients described fatigue as episodic, whereas others described sleep-related symptoms as relentless: “*It’s not like I can just go to sleep and get a real good night’s sleep*”.

Sleep disturbances were reported as causing global weakness and compromised immune function: “*Every time I wind up with pneumonia, I come out a little weaker…Being that I can’t drink or eat anything anymore, all I’m getting is Ensure through my belly…*”, which persisted into a longer-term symptom burden: “*I get weaker all the time. Over the months.*”, and “*Much worse, yes, than it used to be 2 or 3 years ago*”.

Patterns, such as being more fatigued in the morning, or related to exertion, were reported: “*The harder I do anything, or try to do stuff, the more it wears me out*”. 

In addition to fatigue, participants described impediments to falling or staying sleep, poor sleep quality, and undertaking modifications to promote sleep: “*I don’t sleep all the way through the night. When I go to sleep, I probably sleep for four or five hours. Then I’ll wake up*.”, and “*I used to be able to sleep through the night. Now I sleep [inaudible 00:15:53]. I’ll sleep for an hour or two and then I’ll wake up and [inaudible 00:16:03] four or five, and then I’ll sleep for another hour or two and that is the way it is*.” 

Others described sleep–wake cycle derangements and making modifications to bedtime routines, although not always with success: “*Then, there are times, generally, I average about two hours. During the night I’m up going to the bathroom… There have been times when I’ve gotten up every 45 minutes. That’s when it’s really … I’m just out for the next day.”*, and “*I put a fan where it will blow on my face. I learned to put a wash rag in a Ziploc bag because I know the more you get up the less you’ll sleep. If I get to being really bad, I’ll take the cold wash rag and lay it on my neck on the side of face, facing the fan and then I will go back to sleep*”.

### 3.4. Medical Condition

The fifth most common chronic burden pertained to the development of other medical conditions. Aerodigestive tract damage during disease and/or treatments frequently caused longer-term medical complications for patients, including recurrent pneumonia and difficulty breathing: “*When we first went on the feeding tube because the aspiration pneumonia we, he was getting it twice a month…*”, “*I have pneumonia on a very regular basis because food goes the wrong way, if I did use food. Now I’m on a feeding tube and it still goes up and gets in my lungs. I’m just now getting over pneumonia again. I’ve been in the hospital six different times, not counting the times that I got it caught early enough that I didn’t go*.” Example of difficulty breathing; “*It’s a very [inaudible 00:10:16] process I have to do in order to try to open my nasal passages. I actually have to stick my fingers up in my nose and tear out that crusting in order to be able to breathe and to feel better*”.

Medications for anxiety, sleep, and pain caused contraindications for some patients, to blood pressure, physical ailments, weight changes, and addictions: “*I have hypothyroidism from the radiation [inaudible 00:03:29]. I’ve had several episodes of hypoglycemia during treatment. High blood pressure, high cholesterol, all the stuff that goes with it*”.

Patients discussed experiencing co-occurring physical conditions, involving autoimmune, endocrine, heart, and other chronic complications: “*Yeah, the steroids induced diabetes so that’s been another fight…*”.

### 3.5. Cognition

Cognitive symptoms, involving concentration, executive functioning, and memory, were the sixth most discussed in 13 patients. Short-term memory was the most affected cognitive domain: “*I don’t know. They just keep getting worse, nothing … I can remember things happen 20 years ago, but I can’t hardly remember what happened yesterday, you know what I mean?*”.

Executive dysfunction was also reported, for example: “*My mind’s not as sharp as it was…Sometimes I have trouble formulating a full sentence to come out of my mouth.*” “*I would catch myself trying to, thinking of the words, what do I need to say in my mind before. I try to grasp my words before, in a conversation, such as this*”.

### 3.6. Interpersonal Relationships

Significant detriment to relationships was common (ranking seventh). Loss of interest in pursuing connection with others and isolation was reported: “*Yeah, it’s affected all the relationships, everybody in my life. I’ve become a recluse… I’ve lost respect from my parents and my friends and family and pretty much everybody I know.*”, and “*It makes me feel kind of alienated from those people*”.

Some patients perceived a lack of empathy from close ones, which further strained intimacy, connection, and feelings of self-worth “*I think terribly. At one point my husband didn’t understand it at all. This whole thing is a nightmare. Some people they understand it but they don’t quite get it until they see me in action*”, and “*Most of the people I knew at the time I got sick are not in my life anymore*”.

Long-term aftermath affected romantic relationships: “*Sexual drive is completely gone. I had sex one time last year, none this year, which affects relationship, obviously.*” and “*…you haven’t had sex in a year and your wife’s upset about that*”. The effects of chronic symptoms degraded relationship quality with others, sometimes contributing to divorce: “*Me and my wife had been together for like eighteen years. I didn’t want to lose my wife. That happened*”. 

Less commonly, patients reported a transformative experience that increased social support and closeness with others: “*we have a tremendous support group at Guilders club*”, and “*[family, friends] accept the fact that I’ve had cancer and whatever they can do to make my life easier they want to help contribute to that*”.

### 3.7. Pain

Pain symptomatology, the eight most common burden, encompassed localized head/neck pain, muscle, joint, and general aches and pains. A majority of patients discussed acute pain of the throat, neck, tongue, and/or mouth: “*This burning sensation in my throat and in my stomach, it was burning so bad. I don’t know what a heart attack feels like but when I say severe pain, the pain was so severe that I was threatening, a couple times, to go to the hospital”.* This developed into a chronic condition for many patients: “*I feel like I’m supposed to be healing and it seems like I’m getting worse…the pain is real, it’s very real*”; another patient: “*I have significant pain in my neck but it’s not unbearable pain. I can’t say that I’m more uncomfortable. It’s nothing like the pain with radiation…*”.

Other localized head/neck pain constituted chronic headaches: “*I’ve got a constant headache.*”; another patient: “*Headaches. Lots of headaches. Lots of headaches*”.

Muscle and joint pains were also a recurrent physical complaint: “*…the muscle spasm and the pain has come back. The muscle spasms it’s consistent now. I’m not saying every other hour. This is like every five or 10 min. I could be doing nothing, all of a sudden it’ll jump up in my neck.*”, and “*My neck cramps. I’ll get muscles … I call them muscle spasms. I’ll get those in there quite a bit. I’m constantly having to stretch my neck to stretch things like that out*”.

For some patients, long-term physical pain had ultimately spread throughout the entire body: “*I ache all over.*”, “*Widespread aches…*” “*I know my body hurts more… Some mornings, I wake up first thing in the morning, and I just sort of hurt all over. I really can’t move*”.

### 3.8. Appetite and Food

Weight gain/loss, increased/decreased appetite, hypogeusia, dysgeusia, ageusia, and severe difficulties with eating/drinking with the ninth most common burden for patients’ quality of life: “*I think the main thing … I would guess probably one of the bigger issues was my taste. Now I do have the little swallowing, that little flap that covers your windpipe probably*”. 

Choking and difficulty swallowing was reported by many patients: “*It’s weird everybody looking at you. That’s just like when I go out and eat, I still tend to have choking spells. Today I had a little one at a restaurant. I thought half the restaurant was going to get up and give me some, what is it? The Heimlich Maneuver*”.

The use of feeding tubes was also commonly discussed as causing various complications with eating and appetite, and some patients reporting that they received little if any psychoeducation with regards to these issues that would afflict them long-term: “*They didn’t tell me anything about that at the time. No support, no nothing, and I didn’t realize until after four years later. Going through chemo and radiation, I was drinking all that Ensure, which is full of a lot of sugar and all that soy protein isolate and stuff, so basically what I was doing was feeding a whole colony of gut bacteria off of sugar and things. When I changed my diet last year, I reestablished some good [flauna 00:07:23], and that turned a lot of things around*”.

### 3.9. Body Temperature

Metabolic dysregulation, such as feeling overly hot or cold, and experiencing chronic hyperhidrosis, were reported globally: “*I’m telling you I got cold like a dead man… I had come in there with multiple, 3 layer clothes on. Multiple blankets wrapped around me. Get in a wheelchair and then add 2 or 3 heating blankets, I still couldn’t get warm*”.

Sometimes temperature dysregulation was localized, and could be pinpointed by survivors: “*…it’s the extremities. I go numb, my fingers and my toes get numb, for no reason at all. It’s warm outside, but my fingers are numb. My toes are numb. I seem to get cold quicker. A lot quicker*”.

These metabolic issues effected other areas of patients’ lives: “*That first summer I wore winter clothes. I had to go out around everybody and I shouldn’t really care. I don’t in a way care what anybody thinks. It is weird going out like that. It’s weird everybody looking at you*”.

## 4. Discussion

**Figure 1:**
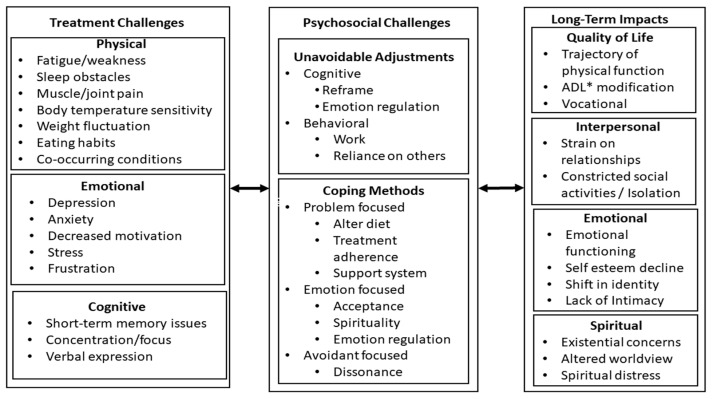
Classification framework of bio–psycho–social dimensions associated with the cancer trajectory and cancer survivorship. * ADL = activities of daily living.

To the best of our knowledge, this is the first in-depth qualitative study to characterize chronic systemic symptoms in cancer-free head and neck cancer survivors with a significant long-term symptom burden ≥12 months from treatment completion. In sum, it highlights that (i) following treatment cessation and the eradication of the disease, a new subset of symptomatology is presented in some patients; (ii) such symptoms are frequently reported in clusters and/or deteriorate into global dysfunction, often requiring long-term care; (iii) aside from the disparate onset of interdependent symptomatology, patients may experience a fundamental transformation in self-concept that is not addressed in the clinical literature; and (iv) currently there is a lack of knowledge, data available, and clinical support for patients experiencing chronic systemic symptoms. Interestingly, the most commonly reported long-term burden pertained to mood dysfunction, alongside symptoms affecting daily activity and fatigue, comprising a primary symptom cluster. This aligns with a systematic review of symptom burden in cancer survivors among the four most common cancer types (breast, gynecological, prostrate, and rectal/colon), exposed to different treatment modalities [35]. Across the longitudinal and cross-sectional assessments, depressive symptoms, pain, and fatigue were most commonly reported and could be experienced by all cancer survivor types for >10 years following treatment/remission [35]. Our qualitative data suggest that mood disorder and interpersonal symptoms (encompassing professional remits, social connection, and intimacy) may be epiphenomenal to the significant disruptions to central physical and cognitive functionality. However, patients are better able to implement adjustments for the latter, reflected by nearly half of all the collected interview data describing some form of emotional impairment significantly eroding quality of life. The physical symptoms of head and neck cancer survivorship have been widely acknowledged/reported. For example, an international cross-sectional study [“Late toxicity and long-time quality of life in HNC survivors”, EORTC 1629 project] assessing long-term toxicities and quality of life in head and neck cancer patients, 5 years post-diagnosis, reported dry mouth and difficulty swallowing or speaking as the most frequent symptoms [36]. The study methodology was quantitative, posing one question in the patient’s case report form that asked for the two main serious effects presently being experienced. It may be that physical (dys)functionality is expected and accounted for by clinicians, whereas efficient aftercare needs to further integrate appropriate psychoeducation, emotion, social, and spiritual support. We outline these dynamics in an overarching framework as a pathway to qualitatively classify germane dimensions of the bio–psycho–social trajectory across cancer control to survivorship (see Figure 1).

One extending factor to consider with the aforementioned framework is that many of the dimensions classified are intrinsically linked to “cultural boundaries”, highlighting the importance of cultural competence in survivorship care. Patients from diverse backgrounds may have unique cultural beliefs and practices that influence their experiences of chronic symptoms, and thus the long-term impact and their preferences for ensuing care. Presently, scant data are available regarding the effects of race, ethnicity, or other cultural identities upon the survivorship experience. However, examination of the Childhood Cancer Survivor Study—an extensive longitudinal research endeavor tracking more than 10,000 survivors of childhood cancers at 5 years+ in remission and their sibling controls [37]—showed that adjusting for socio-economic status did *not* affect health status or mortality rates across white, black, or Hispanic populations [38,39]. However, the original study reported (back in 2005) that minority cancer survivors were more likely to have lower socio-economic status, and when adjusting for income, education, and health insurance, Black survivors were less likely to report adverse mental health [37]. We surmise it is highly likely that these early data reflect cultural perspectives towards mental health and stigmas, biases in reportage, and barriers to accessing mental health cancer supportive care. As such, clinicians and healthcare providers must be sensitive to these cultural factors, read between the lines, and work collaboratively with patients (and their communities) to develop personalized care plans that respect a diverse range of cultural values and worldviews, whilst providing optimal survivorship intervention/s. This extends the entire cancer trajectory to include an inclusive/collaborative approach with patients (and their loved ones) when discussing diagnosis and treatment options, and the provision of resources/psychoeducation, so that patients are as informed as possible throughout the entire cancer process. This will facilitate a smoother transition into survivorship, ultimately ensuring unbiased care for all. 

Looking forward, leveraging technology presents one potential solution to bridge gaps in care and promote inclusion for head and neck cancer survivors from diverse backgrounds and catchment areas that face unique challenges. As smartphone ownership/use becomes ubiquitous at a global scale [40], digital healthcare solutions can facilitate accessibility to culturally sensitive and personalized care for all patients, irrespective of socio-economic status or geographical location [41]. Embracing these technological advances can usher in a new era of equitable survivorship care for the cancer community as a whole, including the unique challenges experienced by the head and neck cancer population. This approach has particular promise for overcoming the aspects of social anxiety and related interpersonal issues reported here by head and neck cancer survivors, through the use of immersive interactive technologies [42]. As our qualitative study highlights, issues with swallowing, speech, facial disfigurements, and changes in appearance can have knock-on effects upon psychosocial domains, such as self-esteem and social interactions. These physical changes may lead to feelings of self-consciousness, social isolation, and even discrimination, significantly impacting quality of life. One innovative solution draws upon emerging technologies such as virtual reality and augmented reality platforms [43,44], which can provide head and neck cancer survivors with immersive experiences that empower them to navigate social interactions with confidence. Through virtual simulations, survivors can rehearse real-world scenarios, such as job interviews, social gatherings, or public speaking engagements, in a safe and supportive environment. These simulations allow survivors to overcome social challenges while honing communication skills and rebuilding their self-assurance, esteem, and self-concept. While not new per se, telemedicine and tele-rehabilitation services [45] have become more sophisticated, enabling head and neck cancer survivors to access specialized care and support remotely, improving healthcare accessibility. A scoping review of 59 studies in long-term survivors (1 year+) reported that overall cancer survivors were satisfied with telehealth interventions, although improving comprehensibility, personalization of platforms, and information accessibility were also highlighted [46]. Remote monitoring tools, wearable devices, and mobile applications also offer personalized health tracking and self-management resources, empowering survivors to actively participate in their care and monitor their progress between medical appointments, especially as they become sparser further out from cancer remission. Minimal research has been conducted on survivorship, although integration of monitoring devices for cancer patients during their radiotherapy course has shown their feasibility, patient engagement, and compliance [47]. The integration of technology into survivorship care programs will facilitate healthcare delivery of tailored interventions that address the comprehensive needs of head and neck cancer survivors, enhancing physical and psychological well-being whilst also promoting social connectedness, personal empowerment, and resilience in the face of significant adversity. Further research and investment in these technologies are essential to ensure that diversity and inclusion are central principles in the future of survivorship care.

Another underdeveloped domain highlighted from our qualitative process is that of spirituality, representing both an area of challenge as one’s worldview shifts following cancer diagnosis/treatment, and also a part of the solution as a pathway of transformation for some survivors. A focus on spiritual needs is often most prevalent in end-of-life/palliative care [48]; however, there is minimal inclusion of spiritual needs in supportive care plans for those with many years of life ahead due to the advances in cancer control. Despite having a profound impact on well-being, spiritual needs of head and neck cancer survivors are often overlooked, and are not integrated into holistic aftercare plans. Practices such as mindfulness, meditation, prayer, yoga, and expressive arts therapy (to name a few), may provide survivors with opportunities for reflection, self-expression, growth, and expansion into existential questions that can aid resilience, cognitive/emotional flexibility, feelings of connection, and overall enhancement of quality of life post cancer. Bridging this gap in supportive care acknowledges the multi-dimensional nature of cancer survivorship. It is also compatible with technological delivery, as head and neck cancer survivors can access spiritual support through apps, online groups, and potentially in the future virtual/augmented reality akin to “immersive spirituality” [42]. A recent assessment in cancer survivorship conceptualized “spiritual distress” as finding meaning/purpose in one’s life, and feeling “connections with a higher power, nature, the world, humanity, or a religion” (or lack thereof) [49]. Notably, the meaning-making process in cancer survivorship has also been highlighted previously within a stress-based framework as an important factor related to survivors’ spirituality and quality of life [50]. The authors of a recent assessment of spiritual distress suggested that clinicians need to be trained in identifying, acknowledging, and providing for spiritual distress by offering evidence-based strategies to mitigate the symptomatic impacts. A consensus forming among clinicians is that nurses on the ground (so to speak) have an optimized opportunity to assess spiritual distress, and may play a pivotal role in spiritual management for cancer survivors across their remission duration [50,51].

### Limitations

Demographics were ethnically homogeneous, and further study is warranted to fully characterize the experience of late head and neck cancer survivors for ecological validity. The participants were disproportionately male, and while this discrepancy was similar to that which exists in head and neck cancer incidence rates in North America, investigation among female-identifying or gender non-binary individuals may further characterize the experience of head and neck cancer survivorship in the broader population. Moreover, we acknowledge that a sample size of 15 may be a limitation. However, it is important to highlight that qualitative research aims to explore in-depth perspectives, experiences, and nuances through a process of unpacking the richness and complexity of the data obtained from a relatively smaller number of participants, as compared to quantitative studies, for example. Finally, patients had to be able to phonate for interviews; thus, modifications needed by all head and neck cancer survivors with regard to language or communication may not be fully represented.

## 5. Conclusions

Head and neck cancer diagnosis and treatment are life-altering. Whilst cancer outcomes improve, acute and late treatment impacts remain detrimental to many survivors. Various coping strategies used to deal with the horrors of the disease and treatments are adopted by patients. However, long-term functional, emotional, and interpersonal alterations often serve to degrade overall quality of life. Development of targeted and multi-level treatment programs that encompass physical, psychosocial, and spiritual domains is needed to increase clinical specificity and effective, integrated, and holistic long-term head and neck cancer survivorship care.

## Figures and Tables

**Table 1:** Mean percentage each theme was discussed across all interviews.

Category	N	Mean % of All Data/ Mean % (s.d.) Weighted for Individual Quotation/Data Contribution
Emotions	15	43.36/36.85 (20.35)
Daily Activities	14	32.16/28.30 (11.39)
Fatigue	14	31.64/28.76 (17.97)
Sleep	15	25.83/21.00 (13.19)
Medical Condition	14	21.89/18.67 (15.19)
Cognition	13	20.02/17.45 (15.79)
Interpersonal Relationships	13	19.19/17.50 (12.21)
Pain	14	18.46/16.54 (9.89)
Appetite and Food	15	17.63/18.08 (8.24)
Body Temperature	14	14.83/14.14 (9.13)
Coping Strategies	7	6.95/5.75 (7.06)
Symptom Improvement	9	6.02/4.92 (5.12)
Disability	8	4.56/4.30 (6.47)
Declining Trajectory	5	2.90/2.59 (4.79)
Delayed Symptom Onset	5	2.28/1.73 (3.95)
Spirituality	4	2.18/1.78 (3.61)

**Table 2:** Exemplary quotations across the entire cohort for the main themes discussed, and comprising at least 10% of all quotations.

Theme	Quotation
**Emotions**	I get to worrying. I start to stress. Yeah, I’m going to admit that I have said to myself, it’s like, “Okay, you’re three years out, you should be getting better.” Then this negativity comes in my mind, it’s like, “Is there something else going on?”
	The depression I think is from the weariness of never being able to escape from the process of symptoms.
	It’s like it was a chemically-induced depression, and I think looking back, it probably was just the hormone imbalances that all occurred. I’ve worked through those. I don’t think that depression exists in my life today because of the chemical imbalance. I think that any depression I have today is because of the hole I’ve gotten into from not working for four years, and I have hope and I can get out of that mess.
	The anxiety, like something upsets me, I used to get upset and speak my mind and whatever and get over it. Now if I get upset, I’m really upset. I can get upset over something that upsets 3 or 4 days which is not normal. I mean highly upset. Sick to my stomach, can’t sit down, can’t think, really upset.
	When I first got out of that treatment, I could not go in the grocery store. It upset so bad I couldn’t. I’d lose it in a grocery store. I still won’t stay in there very long. You know how you usually go around, you look, you got your list and you look and all and things. Well I could put this. I don’t do that anymore. I run through the grocery store like I’m, uh. I don’t know. It’s like I’m running for my life probably. That’s what it looks like. I probably look like an idiot. I hate that. What bothers me about this whole ordeal is that I’ve always been the one that it doesn’t matter what happens or what comes up, I’m very strong willed. This has left me not that way so much. It aggravates me. I would like to think I used to when I got my back up against the wall, I’m going to fight. I’m not a runner. Now it makes me feel like I’m a more of runner. I’ve never liked that feeling. I want to be a stander and a fighter. It takes that out of me and I don’t like it.
	It just aggravating more than anything. It’s aggravating and I don’t know that it affects me too much one way. It’s just aggravating me. It aggravates me is what it does. A lot of this stuff aggravates me now. Especially when I’m having a really bad day. I have really bad days and I have really good days. I rarely have in between days if that makes any sense.
	I think as the time went on and especially when I started losing weight, because at one time, in my mind, I wanted to just give up. I didn’t want to do this no more. Then the thoughts of, especially with my children, I’d get so stressed that I actually I pray all the time but I’m afraid even more, then, the thought of me not being with my children. Yeah, it really took me down through there. Even now those thoughts of if it comes back will I survive this time.
	I went four years with no energy and unable to do any … I was not working at all. You couple that with the chemical imbalances and the depression from that, I’m surprised I’m still married.
**Fatigue and Sleep**	It never went away. I was fatigued from the get go. They said if I have the treatment, the radiation makes you tired. I slept at the first … For 6 months, I slept almost everyday, all day long. All day long. I hadn’t taken anything. I just sat around sleeping. I got over that and I noticed the fatigue. The fatigue really started probably about 6 months out.
	I would say, I’ve got 2. I have this horrible fatigue. I’m fatigued all the time. Then I cannot sleep. I am trying to get off the sleeping pills, but I can’t sleep without sleeping pills, so I have horrible fatigue. The fatigue gets worse.
	Then once I go to sleep, here goes again. I know as soon as go to sleep, I’m going to wake up fatigued. Actually that is what happens.
	I’ve got fatigue so I’m not energetic enough to go do that stuff that she does in Haiti. Yeah. It definitely affects my relationships. Absolutely. I can’t go do things with my son. He’s fourteen now. Over the years he’s wanted to do things. I just didn’t have enough energy to do them. You know?
	I think it causes frustration in me because I can’t figure out why I can’t sleep. I’ve tried meditation, I’ve tried a dark room. I’ve tried everything I can think of. I do everything they tell me to do. I’ve tried melatonin. Those things are a joke, compared to the insomnia that I’m talking about.
	Very difficult time falling asleep. Even at that, my nose is always hurting me. It’s bothersome. It gives me, for some reason and I don’t know why, it just could be just because I’m trying to go to sleep, but it gives me particular fits around bedtime.
	I had to depend on anybody and everybody to do anything for me. Even feeding. I had a feeding tube. I had somebody to feed me everyday. Basically, from that point right there, trying to rebuild myself, I’ve come a long ways, but from that point, it has been a struggle to … I can just as easily, I guess, sit down in the chair and not do nothing than I can to get up and make myself to go do something. It’s a constant mind game with me to tell me to get up and go, get up and go. You can’t just sit here. You got to get up and go. You can’t just lie down and die, basically.
**Daily Activities**	Then my hobby I decided that I had to use that up, and so I get involved in knitting, and I knitted a lot, and now my hands are comprised, so I’ve doing coloring, and now they cannot even color so much. I can color a little bit, but so I’m constantly having to reinvent my hobby. Reading was a hobby. The reason I bring that up and because of the losses then you have to you refuse to be a person who does nothing, or you find new hobbies and its fun the first program or two, but it’s getting gruesome to have to find new ones.
	Not really, but it keeps me reserved in where I’m at at work. You know, I might be more apt to get somebody else to do something instead of doing it myself. I might figure out different ways to get things done than what I used to when I would just do stuff myself.
	It does prevent me sometimes from going and doing stuff with people because I am tired. You know, I’ve exhausted myself all week long or something, and all I want to do is rest a little bit. It does, but I don’t think it does to just a huge amount.
	Like I said, I just lay around about 20-something hours a day. Get up and wife drives me to pick up medicines or go to doctor and that’s about my lifestyle.
**Medical Condition**	If I take more hydrocodone than it should. I have a little withdrawal the next day from some of that. I try not to take the, I like having it but as far as oxycodone, there’s no such thing as a free lunch, that’s a way to put it. If you drink you’re going to have a hangover the next day. It might be fun the night you did it. A lot of that is like the pain medicine too. You’ve got to pay for it the next day and you have to decide what’s worth the price.
	My blood pressure goes to the bottom or out the top. They say I done something to it where it stay very low where I almost pass out and sometimes it’ll go up to 180–90 or 200 on the other end. That’s the start of it. What else you need to know?
	As long as we’re overall recuperated from the smoking part of that to now being damaged by the pneumonia over and over.
	It’s acting up. My nose crusts so bad that sometimes I can’t breathe through it. That’s how bad that gets.
	Yeah. I think I’m on the right path now, just figuring out what it is and the HPA axis dysregulation thing, I think that that’s at the root of the insomnia and the fatigue. The other thing that my wife … she’s standing here … just mentioned another symptom would be apathy, which is complete apathy. When you haven’t paid child support and have to go to court for that and you’re losing your house and you haven’t had sex in a year and your wife’s upset about that, and you haven’t worked and your wife’s upset about the money, it’s like you don’t care. You care, you want to do something about it, but you just … so apathy.
	I’m not generally going to get up the next day and feel pretty good. There’s a big difference if I don’t take the Androgel. Sometimes I forgot too, nobody wants to feel it. In fact, I called Barbara yesterday about Androgel and she gave me another prescription. Possibly forcing that I have so many endocrinological problems that I think Dr. [redacted] is fixing to take me as a primary care patient.
	I have hypothyroidism from the radiation [inaudible 00:03:29]. I’ve had several episodes of hypoglycemia during treatment. High blood pressure, high cholesterol, all the stuff that goes with it. But overall, I feel like I’m doing very well.
**Appetite and Food**	They didn’t tell me anything about that at the time. No support, no nothing, and I didn’t realize until after four years later. Going through chemo and radiation, I was drinking all that Ensure, which is full of a lot of sugar and all that soy protein isolate and stuff, so basically what I was doing was feeding a whole colony of gut bacteria off of sugar and things. When I changed my diet last year, I reestablished some good [flauna 00:07:23], and that turned a lot of things around.
	I tell you, no one really has asked this and I’ve mentioned a couple times. Let me get into this. The cancer I had is a very large cancer at the base of the tongue with a bit at the neck. Anyway, at the time I presented, I was having trouble breathing at night. Got worked up for CPAP, got that started wearing that. When they did the radiation, then it fried a lot of the muscle as well as the tissue of the tongue back there so there’s a divot. Food gets back there and it’s pretty disconcerting. The message that your brain gets is you’re fixing to choke.
	It makes me feel sick really. I’m not embarrassed over it because I’ve never been a big eater. I’ve always been a picky eater. I’m not embarrassed or anything. I wish I wasn’t that way and it’s inconvenient. It’s a big inconvenience is what it is.
	When I left that last radiation treatment. I thought my throat’s killing me. They about cooked it. My mouth’s killing me. I had to keep pain patches on. Once I kicked those pain patches, I’ll be fine. No, I’m not. That’s it. Primarily is the fatigue, the sleeping and I have memory loss.
**Pain**	I have deterioration to the hinges of my jaw especially on the left-hand side from the radiation and I cannot open my mouth very wide.
	Especially around my neck and shoulders. It’s constant muscle spasms. They get so bad, not only with the pain but with the muscle spasms, I can hold my neck, turn my neck, it doesn’t matter. The muscles get so contracted that they actually lock and I have to kind of move my neck in order to free it up. Just like today, which is not the first day, it’s often underneath my neck. The muscle spasm will come. I actually feel the muscle, how big it is, under my neck. Once I move it I can actually feel it go down.
	My neck area, where a lot of the radiation was, where most of it was, it’s got a weird numbing feeling to it. The side of my neck all the way up to like my ear and going up the side of my head a little bit, it has just like a weird type numbing feeling to it. The left side of my neck and head does not feel nothing like what the right side does. It doesn’t hurt. I mean, it’s not that uncomfortable. It just feels different. It just feels like it’s got a little bit of that … You know how … Well, it’s not the tingling thing that you get in your fingers, but it’s some kind of a numbing type. It just feels like you don’t have all the feeling in there.
	The pain? Or aches and pain? Oh yeah. It’s all over my body. It’s my muscles. It’s not joints. It’s worsened.
	I know my body hurts more. My knees hurt more. Elbows and joints and stuff like that. They just overall … Some mornings, I wake up first thing in the morning, and I just sort of hurt all over. I really can’t move. I just have to sit for a little while, and I’ve got a little claw that’s like a little massager thing, and I’ll just put it on my neck and sort of massage my neck and my shoulder and everything from where the radiation was. They told me one time that lymphedema, maybe, I think is what they called it. That my neck, I’ll wake up, and my neck’ll be really hard, and it’ll be swollen. I’ll use that and sort of relax everything, sort of get everything to moving, and it just takes my body awhile to get into a groove into moving. I mean, it’s a struggle sometimes.
**Cognition**	My short-term memory’s not good at all. I can pull things back to medical school, that ain’t a problem, or even before…But thinking about something, I have trouble driving and keeping between the lines and not having an accident. I’ll miss turns, so I have to really concentrate. I’ve got where I have trouble remembering routes to certain places. So I’m using technology more often. I go to a parking lot. I can lose my car fairly easily. So whenever I started driving again, I didn’t drive for over a year, I’ve got a red Cadillac Escalade. I can see it half a mile. There’s things that I’ve done to minimize the, not the symptoms, but the outcomes.
	Yeah. [inaudible 00:23:00] and that sort of thing. A lot of times now I have trouble spelling words that I know I know how to spell.
	I’ll stammer around looking for a word or something. You know, those are things that I’ve just experienced that I’ve never had a problem with before. I guess it’s formulating that full thought process and letting it come out of your mouth, I frustrate myself because it doesn’t want to do that a lot.
	It’s primarily cognition. I don’t think I can work as a surgeon again because the tremors have gotten worse. I get patients mixed up and it wouldn’t work out. I thought I could come over to the cancer clinic. As a physician, after having the radiation, I might have something to say to somebody is my thought process but that’s the way it’s going to work out.
	Yeah, I just had no focus or concentration. I talk really. I still read and one thing I noticed that I can’t even read when they put subtitles up on T.V. for what tutors is saying. I can’t read them before I go away. My reading has slowed down tremendously, and I don’t understand that at all but I could read a book in a week and it’s just now taking me too much to finish this aggression book. Was it a good thing but to my reading comprehension and focus and concentration are gone, and my attention to detail is not as good. The attention to detail is not good.
	I mean, because people want to say, ‘Well, you’re 3 years older.’ Well, you know what? That’s BS. I know what I was. I know what I am now, and there is a pretty good difference in the two… I know what it’s done … what it’s left me being. It’s not the same person that I was. My mind’s not as sharp as it was. I forget things that I used to not ever forget, names, people.
**Interpersonal Relationships**	Yeah, it’s affected all the relationships, everybody in my life. I’ve become a recluse, or became a recluse. I’ve lost respect from my parents and my friends and family and pretty much everybody I know. They think this is all something in my head. I don’t want to call out any names. The last doctor I went to was there at your hospital, and they told me that there was something in the brain. It’s like something gets switched in the body and they don’t know what it is and they don’t know how to switch it back. Go buy a Fitbit and sign up for yoga class and find a family member that will support me the rest of my life. I got a bill for $384 for that advice, and I was floored. I was floored by all fourteen [inaudible 00:15:13] doctors that would charge me even though they were not able to help me, so that is extremely frustrating. I guess I should be grateful that the cancer was killed, but I will say that there are probably as many days over the last five years that I wish I had not survived the cancer.
	It effects my relationship with my wife. In any relationship, it’s a give and take. I’ve had to take more than I give sometimes.
	I have more social anxiety, and sometimes I get anxious in social situations…If I’m in a situation in the dinner the table, I’ve got a mouth full of food I’ve been working on for five minutes since somebody asked me a question and everybody is looking at me, and I can’t answer.
	I don’t have the same quality in my relationships. I don’t have the same. Everybody says they feel the same but I don’t feel the same because once again, I feel guilty because I don’t feel like doing this. Or I don’t feel like doing that. I can’t remember to do this, that or the other. It’s negative.
	It’s stopped me from being involved in a lot of other people’s lives, so … I don’t know. I just woke up a little bit ago, so I can’t … just when fatigue, when you’ve got that much of it, I don’t know, it’s …
	Some people, if they don’t see an injury, they don’t understand the pain. They don’t understand that you’re hurting. Unless you have a cast on your leg or arm, quote unquote, but I can be aching and it’s not a visible scar, if you will. They don’t, and I was the same way. I didn’t understand it. We classify people oh they just don’t want to work. Or they just don’t want to do whatever. I understand more now, that’s for sure.
**Body Temperature**	It happened. I guess that on and off thing, get real cold in January after I got treatment. It was January after the treatment. The treatment was 5 October 2013. In January 2014, I got cold. I’m telling you I got cold like a dead man. I immediately went to Vanderbilt. They examined me. I offended a few of the doctors. They called Dr. [redacted]. Dr. [redacted] come in there. I was cold and clammy, not all the time, clammy to the touch. My body temperature said it was about 96, 97 sometimes it would even read normal but I would be terrible cold. Cold like I’m telling you, I don’t even know how to tell you. Dr. [redacted] seen me though. I had come in there with multiple, 3 layer clothes on. Multiple blankets wrapped around me. Get in a wheelchair and then add 2 or 3 heating blankets, I still couldn’t get warm.
	I had to wear a coat in the summer, that summer. I know everybody thought I was a fool.
	Sometimes I have to get a jacket out and that shawl. Every time I go to the hospital up there, I have to have that shawl. I die in that hospital. If I didn’t have that shawl I’d sit there and freeze. No, I react to cold and heat more that than I think is normal. I don’t think that’s normal. I never did it before.
	It’s a little bit embarrassing because I’m the only one sweating and nobody else is, and I wear my hair short now but before I had such fine thin hair that I would have my, throw my hair back, [pasted 00:19:36] down because of my sweat, it affected the way I looked, which in turn affect the way I felt.

## Data Availability

Data will be made available upon reasonable request.

## Supplementary Materials

Supporting information regarding the methodological coding system can be downloaded at https://healthbehavior.psy.vanderbilt.edu//Wulff/Wulff_CodingSystem.pdf (accessed on 17 May 2024).
